# Predicting functional sites with an automated algorithm suitable for heterogeneous datasets

**DOI:** 10.1186/1471-2105-6-116

**Published:** 2005-05-13

**Authors:** David La, Dennis R Livesay

**Affiliations:** 1 Department of Biological Sciences, California State Polytechnic University, Pomona, California 91768 USA; 2 Department of Chemistry and Center for Macromolecular Modeling & Materials Design, California State Polytechnic University, Pomona, California 91768, USA

## Abstract

**Background:**

In a previous report (La et al., *Proteins*, 2005), we have demonstrated that the identification of phylogenetic motifs, protein sequence fragments conserving the overall familial phylogeny, represent a promising approach for sequence/function annotation. Across a structurally and functionally heterogeneous dataset, phylogenetic motifs have been demonstrated to correspond to a wide variety of functional site archetypes, including those defined by surface loops, active site clefts, and less exposed regions. However, in our original demonstration of the technique, phylogenetic motif identification is dependent upon a manually determined similarity threshold, prohibiting large-scale application of the technique.

**Results:**

In this report, we present an algorithmic approach that determines thresholds without human subjectivity. The approach relies on significant raw data preprocessing to improve signal detection. Subsequently, Partition Around Medoids Clustering (PAMC) of the similarity scores assesses sequence fragments where functional annotation remains in question. The accuracy of the approach is confirmed through comparisons to our previous (manual) results and structural analyses. Triosephosphate isomerase and arginyl-tRNA synthetase are discussed as exemplar cases. A quantitative functional site prediction assessment algorithm indicates that the phylogenetic motif predictions, which require sequence information only, are nearly as good as those from evolutionary trace methods that do incorporate structure.

**Conclusion:**

The automated threshold detection algorithm has been incorporated into MINER, our web-based phylogenetic motif identification server. MINER is freely available on the web at . Pre-calculated functional site predictions of the COG database and an implementation of the threshold detection algorithm, in the R statistical language, can also be accessed at the website.

## Background

Due to the exponential growth of genomic and protein sequence data, development of automated strategies for large scale functional site identification is an important post-genomic challenge. Many recent efforts predict functional sites from sequence alone. Strong candidates for functional sites include individual highly conserved positions within a sequence alignment and highly conserved sequence motifs [1-5]. Although attractive due to their relative simplicity, conservation-based approaches frequently result in too many false positives to be satisfactory [3]. Further, sequence regions with significant variability can also be functionally important [6], especially when their composition may define sub-family functional specificity. The Evolutionary Trace (ET) procedure [7], and similar approaches [6,8,9], address this problem by using evolutionary information to identify subfamily conserved, yet overall variable, positions. It has been demonstrated that the ET methods identify statistically significant structural clusters [10] and has subsequently been established in large scale analyses [11].

Recently, we demonstrated that sequence fragments conserving the overall phylogeny, termed phylogenetic motifs (PMs), are very likely to correspond to protein functional sites [12]. We briefly highlight the key results of our previous report here (see Implementation for a technical description of the approach). Despite little overall sequence proximity, PMs are structurally clustered around a wide variety of protein functional regions, including sites defined by surface loops, active site clefts, substrate binding epitopes, and protein-protein interfaces. Ostensibly, PMs identify sequence clusters of ET positions, and, as expected, the results of the two approaches are similar. However, compared to raw ET predictions, PMs seem to be more structurally focused on active site regions. Lastly, we have demonstrated appreciable tree significance of the fragment windows, especially in PM regions, using bootstrap analysis.

In a recent review, Jones and Thornton [13] classify protein functional site prediction strategies into one of two groups: (1) those based on sequence conservation and (2) those based on "feature" (i.e. phylogenetic) conservation. Congruence between phylogenetic and traditional motifs has been clearly established [12]. As a consequence, PMs bridge the two normally disparate groups. For example, in the case of triosephosphate isomerase, all seven traditional motifs are also identified as PMs, including the PROSITE [14] definition of the family. However, due to the large number of clades within some families (e.g. cytochrome P450) there are instances when PM sequences are not overall conserved. This point is in enticing because it suggests that PMs can sometimes functionally annotate regions that traditional methods would ignore. Furthermore, it has been reported [12] that traditional motifs not conserving phylogeny are less likely to be structurally clustered around known functional sites.

PMs are identified using a sliding sequence window algorithm that exhaustively compares the phylogenetic similarity of each fragment window to the complete familial tree. Phylogenetic similarity z-scores (PSZs), which are defined below, quantify the similarity between the window and familial tree. (Smaller PSZs indicate greater phylogenetic similarity.) In our current approach, all overlapping windows scoring past some PSZ threshold are identified as a PM. The PSZ threshold is manually adjusted to define what constitutes a "hit". Automated threshold determination is a difficult problem because no single threshold value is sufficient for every instance. Each protein family requires a unique value to correctly identify a signal (a PM window) from noise. In our previous work, we manually set threshold values where PSZs strongly deviate from all other values. Structural analyses indicate that ideal PSZ threshold ranges between -1.5 and -2.0. In other words, the ideal phylogenetic similarity cutoff for accurate functional site predictions generally falls between 1.5 and 2 standard deviations away from the mean of the PSZ distribution.

In this report, we describe an automated algorithm for determining proper PSZ thresholds. Structural analyses and comparisons to our previous manual thresholds indicate that the approach retains functional site prediction accuracy. The method utilizes significant raw data preprocessing that eliminates unnecessary (redundant) data points. Subsequently, the robust Partition Around Medoids Clustering (PAMC) algorithm is employed to automatically determine the appropriate PSZ thresholds. The implemented approach is computationally efficient and demonstrated to be suitable for large, heterogeneous datasets, which remains a difficult bioinformatic problem [15].

## Implementation

### Phylogenetic motif identification

During PM identification, we mask the multiple sequence alignment by purging all highly (more than 50%) gapped positions. The masked alignment is parsed into a series of sequence fragment windows of fixed width. In this report, a window width of five, which has previously been shown to be most sensitive for identifying functional sites [12], is used throughout. Except for the copper, zinc superoxide dismutase and myoglobin families, which use the same datasets as before [12], all sequences are taken from the Clusters of Orthologous Groups (COGs) database [16]. Only COGs with more than 25 sequences are investigated to ensure proper and significant tree construction. Pairwise tree similarity is calculated using a modified partition metric algorithm [17], which counts the number topological differences between the fragment window and familial trees. Thus, smaller partition metric scores correspond to greater tree similarity. Phylogenetic similarity is measured using z-scores calculated from the raw partition metric distribution. Although not the best alignment method for distantly related sequences, ClustalW (v1.83) alignments are used throughout [18]. Given the similarity within the COG families, ClustalW alignments are satisfactory.

Phylogenetic trees are constructed using the neighbor-joining implementation within ClustalW. Neighbor-joining is a distance-based approach for constructing phylogenetic trees commonly used for bootstrap analysis that requires massive tree sampling [19]. Similarly, due to the large number of window trees required here, the algorithmic efficiency of distance-based methods is necessary. For example, in the medium-sized triosephosphate isomerase protein family, over 250 trees must be calculated. Furthermore, as Kuhner and Felsenstein point out [20], distance-based approaches actually outperform maximum-likelihood methods when applied to short sequences. MINER, our web-based implementation of the PM identification algorithm, is available online at [21]. A standalone version of MINER, implemented in PERL, is freely available to the Academic community upon request.

### Raw data preprocessing

Empirically, our manual assessment of functional site prediction accuracy indicates that all PSZs below χ ≈ -2 should be identified as PMs, whereas PSZ's above -1 should never be considered. In the subsequent clustering step, only scores between -1 and χ are used to define the PSZ. This simplification is taken because the objective of this work is to automatically classify windows *whose determination remain in question*. The significance of the PSZs outside this range is known *a priori*, thus they can be eliminated from further consideration. Clustering of the data points between -1 and χ (termed the "gap") was originally expected to automatically determine the appropriate PSZ threshold. However, several different clustering techniques (hierarchical, *k*-means, PAMC, and expectation-maximization) have failed to provide satisfactory results.

**Figure 1:**
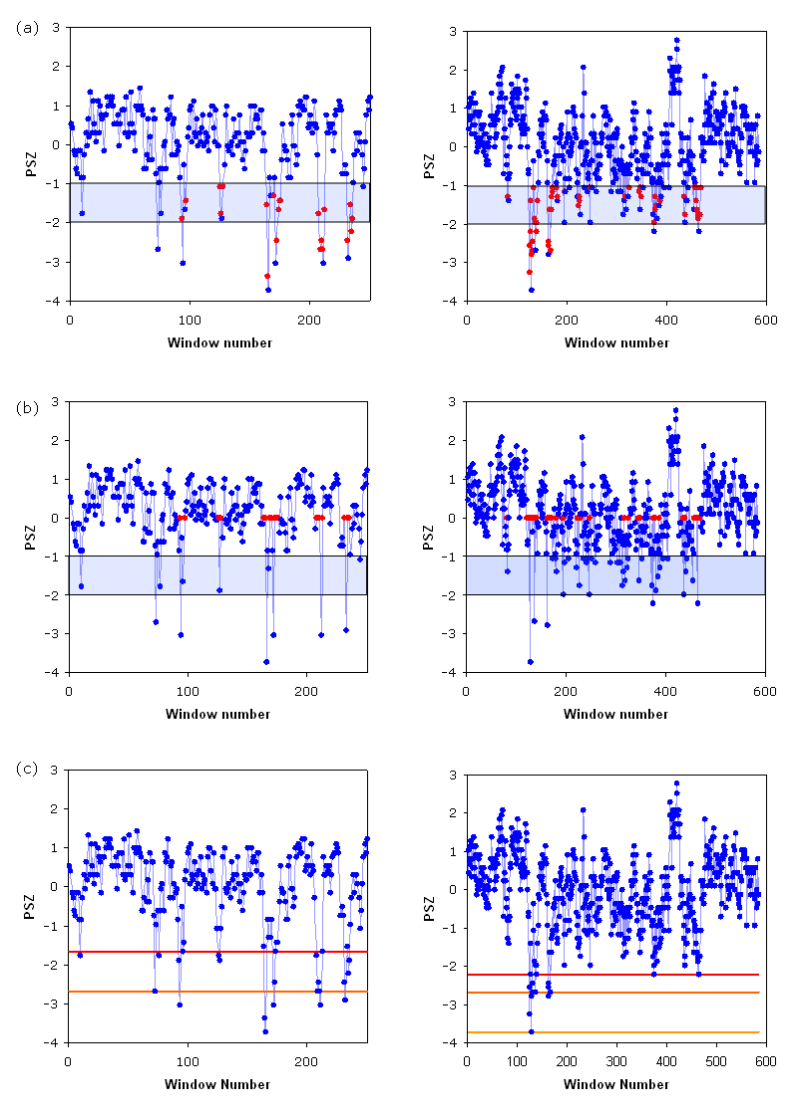
Partitioning Around Medoids Clustering (PAMC) is used to partition PSZs between -1.0 and χ = -2.0 into two groups (signal and noise clusters). The results from the **(A) **raw and **(B) **sharpened PSZ datasets are shown. Red indicated sharpened data points. We demonstrate two common scenarios **(left) **triosephosphate isomerase and **(right) **arginyl-tRNA synthetase. In all cases, it is clear that sharpening the PSZ dataset allows one to more easily discern the number of distinctive potential signals under the partition boundary (gap) in question. **(C) **The effect of different sharpening ranges is demonstrated. PSZ ranges tested include -1.0 to -2.0 (red), -2.5 (dark orange), and -3.0 (light orange). In the case of triosephosphate isomerase the first two ranges give identical results. The ideal threshold is found to be -1.65 and -2.20 for triosephosphate isomerase and arginyl-tRNA synthetase, respectively.

In order to accentuate differences within the PSZ distribution, and thus simplifying the clustering problem, the following preprocessing procedure is employed. As stated above, all overlapping windows scoring past some predetermined threshold are defined as PMs. We use the same rationale in detecting PSZ thresholds. The process begins by identifying all overlapping windows scoring past -1. For the purpose of threshold detection only, we "sharpen" these regions by selecting the lowest window score as a reference; all other scores are eliminated. This process has the effect of reducing the number of contiguous and related PSZ scores (corresponding to overlapping windows) into a single value (see Figure 1). After accentuating the high phylogenetic similarity regions, PAMC can robustly identify the ideal PSZ threshold. Several different upper bounds have been considered, but our empirical results indicate that -1 is best.

### Automated PSZ threshold determination

PAMC is a partitioning algorithm based on the *k*-means approach of clustering [22]. In k-means clustering [23], the center of a cluster is represented by its arithmetic average. In PAMC, each cluster is represented by the median value, making PAMC a *k*-medoids approach. The basic concept of PAMC is to partition a dataset containing a number of points into *k *number clusters. PAMC starts from an initial random set of medoids and iteratively swaps medoids with non-medoids to evaluate if the total distances between clusters are improved. PAMC is more effective and robust than *k*-means for small datasets because medians are less biased by outliers deviating from the mean. Our PSZ dataset is small, especially after preprocessing, making PAMC an appropriate clustering choice. In addition, because the goal is to separate signals (cluster one) from noise (cluster two), k-based approaches are ideal. The preprocessed gap is differentiated into *k *= 2 number of groups. Clustering is performed by the PAMC implementation within the cluster package of the R statistical language [24]. The PAM algorithm implemented in R simply uses the Euclidean measure by default and the Manhattan as a defined alternative. We use the Euclidean measure throughout.

In determining the ideal PSZ threshold value, the number of data points in the signal cluster is counted. If the signal cluster contains five or less data points, the threshold is set to the most accommodating (least negative) value in that cluster. However, an algorithmic override that defines the PSZ threshold at the first (rank ordered) PSZ above χ if any of the following three situations occur: (1) if the signal cluster contains more than five data points, (2) if less than three points reside in the gap – it does not make sense to cluster so few data points into two groups, or (3) no PSZs lower than -2 are present within the distribution. The algorithmic override prevents normalizes the number of putative functional sites, preventing both too many and too few predictions. Empirical results investigating the accuracy of the method's predictions vis-à-vis structure indicate that the algorithmic override maximizes accuracy. For example, in examples where the override reduces the number of predictions, frequently the excluded sites are structurally removed from the active site region. The ideal value of χ is established in the next section.

### Quantitative assessment of functional site predictions

The accuracy of the functional site predictions herein is quantitatively determined using the method put forth by Aloy et al. [25]. In the scheme, a known functional site sphere is defined by the location of SITE and ACTSITE records within a PDB file. In line with our previous report [12], we also include residues directly interacting with substrates and catalytically important metal ions, which are identified using LIGPLOT [26]. Prediction spheres are similarly constructed for each PM. The accuracy of each PM prediction is based on the relative location of the known functional site and prediction spheres. If the prediction sphere is completely engulfed within the known functional site sphere, then the prediction is deemed correct. If the two spheres partially overlap, then the prediction provides useful information. And, if there is no overlap between the two spheres, then the prediction is wrong. In the case of the known functional site, the sphere is centered on the geometric center of the CB atoms (CA for glycine) of all functional residues. Similarly, the PM sphere is centered on the geometric center of the corresponding CB atoms (CA for glycine). In both cases, the sphere is made just large enough to include all functional or PM residues.

## Results and discussion

### Establishing algorithm parameters

As described below, the automatic threshold determination problem is simplified when considering only the most extreme of several contiguous and related, PSZ values. We call this process data sharpening. As a consequence, identification of the true phylogenetic signals is greatly simplified. In contrast, contiguous windows with similar values are the result of a single PM, making it difficult to properly count the number of true signals in an unsharpened dataset. Because PMs are defined as all overlapping windows scoring past the PSZ threshold, reducing the complexity of the problem to be in line with the number of PMs, versus number of windows, makes intuitive sense. For example, if two PMs are considered, the first consisting of three overlapping windows and the second with five, only two unique signals, compared to the eight constituent windows, are considered. Comparisons of sharpened and unsharpened datasets are demonstrated in Figure 1.

Thresholds are determined by first evaluating the optimal range using the PAM clustering algorithm. Determining whether a threshold can be placed within the range of -1.0 and -2.0 allows thresholds to sensitively accommodate more functional sites, widening this range results in more stringent thresholds. Figure 1C illustrates different thresholds determined when considering three different PSZ ranges. By broadening the range of the triosephosphate isomerase (TIM) dataset, two distinct thresholds are found. Ranges of {-1.0: -2.0} and {-1.0: -2.5} identify the same threshold (PSZ = -1.65). However, expanding the gap to {-1.0: -3.0} results in a significantly more stringent threshold (PSZ = -2.86). The former PSZ threshold is more similar to our manual determination of PSZ = -1.5 [12].

Similarly, the arginyl-tRNA synthetase family is evaluated using the same three gap ranges. Like TIM, the determined threshold becomes more stringent as the gap broadens. However, the three gap ranges result in three distinct PSZ threshold values. The arginyl-tRNA synthetase example is noteworthy because the determined threshold in all three instances is algorithmically set below χ. This occurs because too many points exist in the PAM identified signal cluster. Since we assume functional sites cover only a fraction of the protein sequence space, when the signal cluster is larger than five, it is disregarded and the PSZ threshold is set at the first (rank ordered) PSZ past χ.

Comparison of all three gap ranges on the determined PSZ threshold for the 15 functionally and structurally diverse proteins used previously [12] is partially used to determine the ideal gap range. In all cases, the functional significance of the manually determined threshold has been demonstrated using structural analysis. Additionally, the exact catalytic role of many of the identified PMs (especially TIM [27], enolase [28], inorganic pyrophosphatase [29], copper, zince superoxide dismutase [30], and TATA-box binding protein [31]) has also been delineated. For example, eight PMs are identified in the case of TIM, which cover all eight LIGPLOT [26] identified electrostatic interactions between enzyme and substrate. Furthermore, the flexible "lid" region, which covers the active site during catalysis [32-35], is also identified as a functional site. We have also recently demonstrated that PMs within TIM (and two other TIM-barrel families) also correspond to evolutionarily conserved electrostatic networks that fine-tune the pKa values of catalytic residues [36].

**Figure 2:**
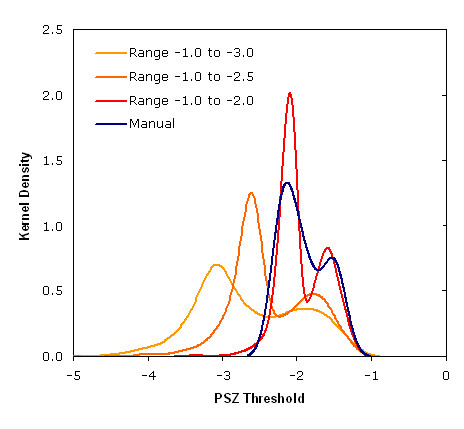
The frequencies of the three different PSZ thresholds are found on 1571 protein families (all COGs with more than 25 sequences). The normalized frequencies are represented as kernel density estimates. All optimal ranges used consist of two maximum densities from -1.0 to -3.0 (PSZ = -3.04 and -1.82), from -1.0 to -2.5 (PSZ = -2.60 and -1.74), and from -1.0 to -2.0 (PSZ = -2.01 and -1.58). Kernel densities from fifteen manually set thresholds that have been structurally verified also contain two maximum densities (PSZ = -2.12 and -1.53). Of the three ranges tested, the {-1.0: -2.0} gap is the only one that is statistically similar to the structurally verified results. The coloring of the three ranges is the same as in Figure 2C.

The frequency of threshold values (displayed as kernel density estimates) determined using the three different gap ranges is illustrated in Figure 2 for the entire COG database [16]. Of the three PSZ ranges tested, the threshold distribution resulting from the narrowest range is most similar to the distribution of our structurally verified dataset. Using a two-sample *t*-test, the statistical significance between the PSZ threshold distributions can be assessed. The t-test results (*t *= -0.41, *p *= 0.69) indicate that the manual and {-1.0: -2.0} distributions are not statistically different. However, when evaluating the manual threshold distribution with the other two gap ranges, (*t *= -6.08, *p *= 2.14 × 10^-05^) and (*t *= -9.49, *p *= 6.85 × 10^-08^) for {-1.0: -2.5} and {-1.0: -3.0}, respectively, we find that the differences are highly significant. Furthermore, the distribution of thresholds from the {-1.0: -2.0} show the most frequent PSZ thresholds are set around -1.5 and -2.0, which is in line with our original conclusions. A gap range of {-1.0: -2.0} is used throughout the remainder of this report. Table 1 compares the manually and automatically determined PSZ thresholds.

### Functional annotation of the COG database

**Figure 3:**
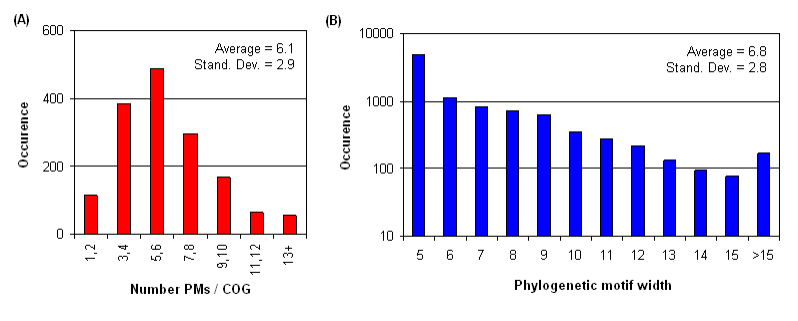
**(A) **Histogram tabulating the number of phylogenetic motifs per COG. **(B) **Histogram tabulating phylogenetic motif width (note logarithmic scale). The theoretical lower bound on motif width (five, which equals one fragment window) occurs 51% of the time. The {-1.0: -2.0} gap range is used in both cases.

**Figure 4:**
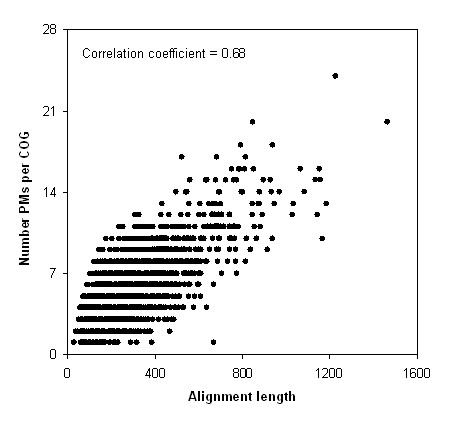
Number of phylogenetic motifs per COG, using the {-1.0: -2.0} gap range, is plotted against alignment length. As expected, there is a direct correlation between the two quantities. At least one phylogenetic motif is identified in each COG. Conversely, in large COGs 10+ phylogenetic motifs are routinely identified.

Using the procedure established above, we exhaustively functionally annotated the most recent update [16] of the COG database. After parsing out COGs with less than 25 sequences, our dataset is composed of 1571 protein families. The number of PMs identified resembles a bell curve centered on 6.1 motifs per COG (Figure 3A). The standard deviation is 2.9. 24 PMs, the most of any COG investigated, are identified within the cobalamin biosynthesis protein family. Due to the extreme size of this protein, the number of identified PMs is within the expected range – the cobalamin biosynthesis protein family alignment is the second longest in our dataset. Consistent with our earlier qualitative observations [36], Figure 4 reveals a roughly linear correlation between alignment length and the number of phylogenetic motifs identified per COG.

In total, 9558 PMs are identified. Compared to the number of PMs per COG, there is much more heterogeneity within the motif width distribution (Figure 3B). The theoretical lower bound on PM width is five (one fragment window); whereas there is no limit on their maximum size. A motif width of five is by far the most common, occurring 51% of the time. The maximum width observed, which occurs in the methyl-accepting chemotaxis protein family, is 42 (occurring once). The large motif corresponds to the chemotaxis transduction 2 domain. As a stark contrast, only one other PM (width = 5) is identified within this family. The second, and much smaller, motif coincides with the PROSITE [14] definition (R-T-E- [EQ]-Q) of the family. The [EQ] position is a site of reversible methylation.

The large-scale nature of this analysis provides an opportunity to assess the dependence of several factors on the automatically determined PSZ thresholds. Correlations between the determined PSZ thresholds and number of PMs identified, number of sequences in the dataset, and alignment length are calculated (see Table 2). As discussed, a roughly linear (R = 0.68) correlation between number of PMs identified and alignment length is identified (Figure 4). However, no other strong correlations are identified between any of the probed characteristics. While more-or-less uninteresting, this result is actually encouraging because it indicates that PMs, in addition to being accurate, represent a robust functional site prediction algorithm suitable for large, heterogeneous datasets.

### Molecular examples

**Figure 5:**
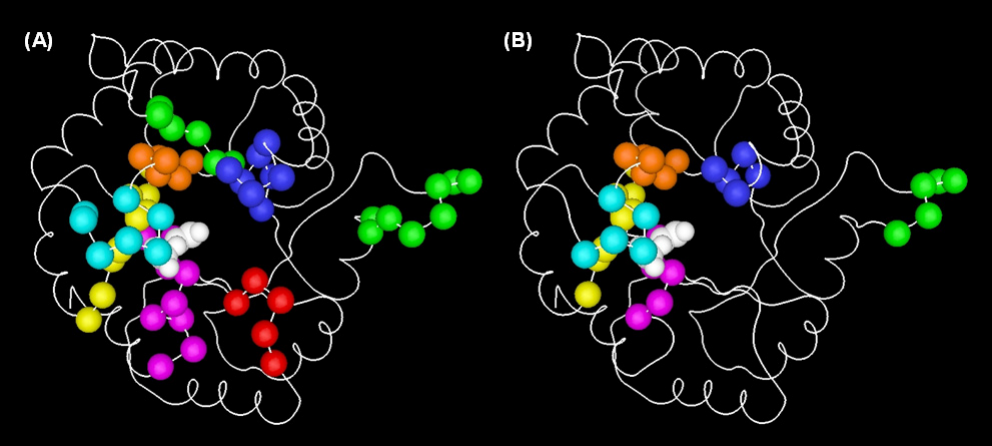
Triosephosphate isomerase phylogenetic motifs identified at the two different PSZ thresholds. Colored spheres indicate phylogenetic motif α-carbons identified past the **(A) **-1.83 and **(B) **-2.75 thresholds, which correspond to gap ranges of {-1.0: -2.0} and {-1.0, -3.0}, respectively. The {-1.0: -2.5} gap range identifies the same threshold as (A). The substrate analog is colored white. Making the threshold more stringent fails to identify residues (the red phylogenetic motif) involved in two stabilizing enzyme-substrate interactions (a salt bridge and an H-bond).

Clustering of the three different TIM gap ranges uncovers two putative PSZ thresholds (see Figure 1C). We demonstrate above that the narrowest gap range (and as a consequence, the most lenient PSZ threshold) to be appropriate. In this case, however, both the {-1.0: -2.0} and the {-1.0: -2.5} gap ranges set the threshold at -1.83. The determined threshold sensitively accommodates the complete substrate binding epitope, including all eight enzyme-substrate electrostatic interactions. However, the {-1.0: -3.0} range identifies a more stringent threshold (-2.75), which misses one enzyme-substrate salt bridge and one hydrogen bond. Several other less drastic differences also occur. A structural analysis of these automatically set thresholds is shown in Figure 5. Despite the differences between the two thresholds, both identify PMs that correspond to the PROSITE [14] definition of the family, the flexible "lid", and most of the enzyme-substrate contacts. In both cases, all identified PMs are structurally clustered at the C-terminal end of the barrel.

**Figure 6:**
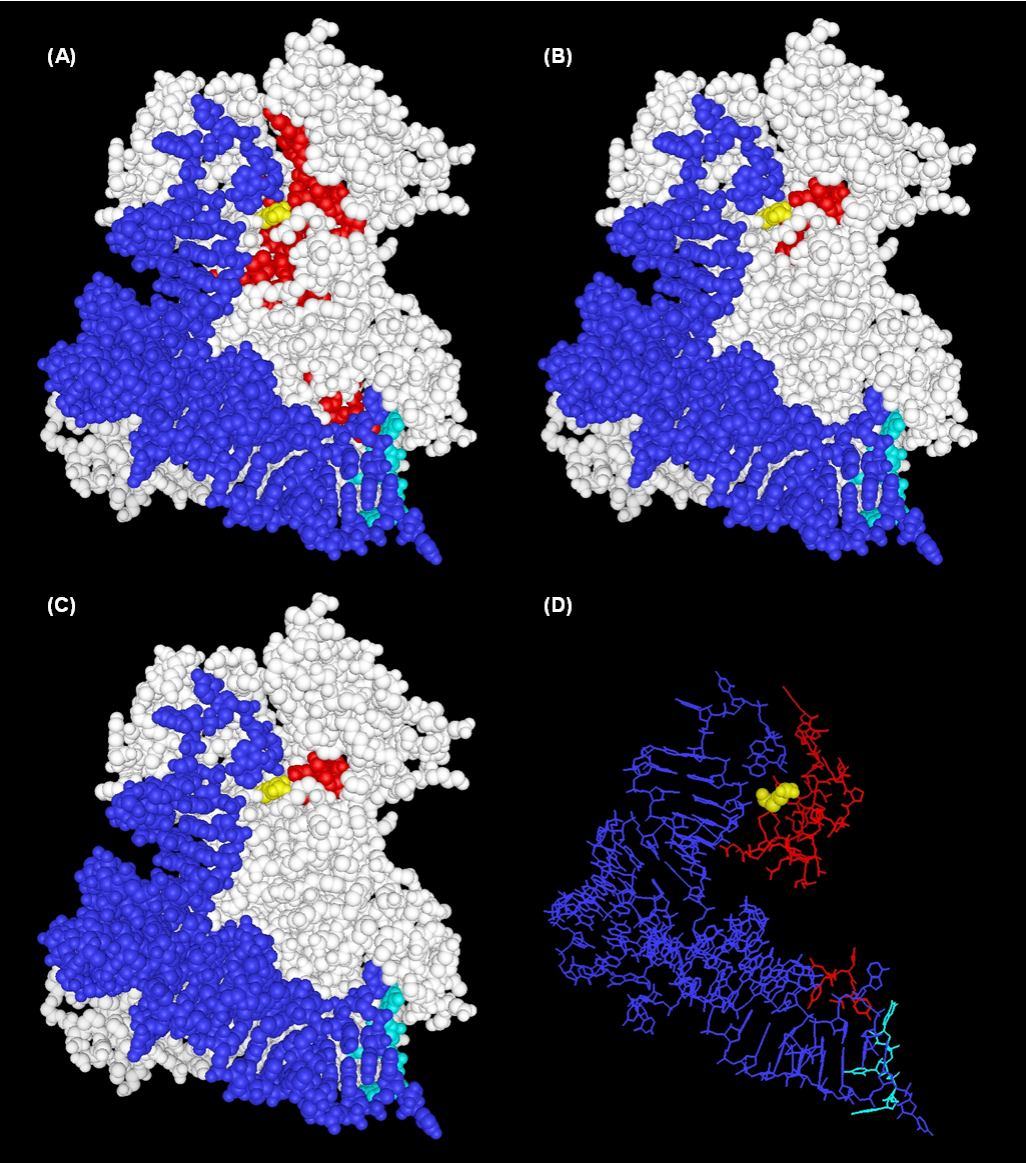
Arginyl-tRNA synthetase phylogenetic motifs at the three PSZ thresholds are highlighted. Residues colored red indicate phylogenetic motifs identified using the **(A) **-2.04, **(B) **-2.93, and **(C) **-3.26 PSZ thresholds, which correspond to gap ranges of {-1.0: -2.0}, {-1.0, -2.5}, and {-1.0, -3.0}, respectively. The arginine substrate is colored yellow; the tRNA is colored blue; and the anticodon is color cyan. Using (A), two structurally unique clusters of phylogenetic motifs are identified. One corresponds to the enzyme active site, whereas the other corresponds to three H-bonds between the enzyme and the anti-codon arm of tRNA. **(D) **is the same (A), but with the rest of the enzyme removed for clarity.

We also structurally verify functional site prediction accuracy within the arginyl-tRNA synthetase family, which is a previously unreported example. As with TIM, we evaluate the same three gap ranges. Structural verification and comparison of these three thresholds is illustrated in Figure 6A–C. The more accommodating PSZ threshold identifies two structurally unique PM clusters. The first is composed of four PMs, and corresponds to the enzyme active site. Several stabilizing enzyme-tRNA and enzyme-Arg interactions are included in this region (Figure 6D). The second PM structural cluster is composed of a single PM, and corresponds to three enzyme-tRNA H-bonds at the tRNA anticodon arm [37]. Making the PSZ threshold more stringent, by widening to the gap range to {-1.0: -2.5}, eliminates two PMs, including the anticodon arm PM. Only one PM is identified at the most stringent level. Like with TIM, the ends of the identified PMs are trimmed at increasingly stringent PSZ thresholds.

### Assessment of functional site predictions

**Figure 7:**
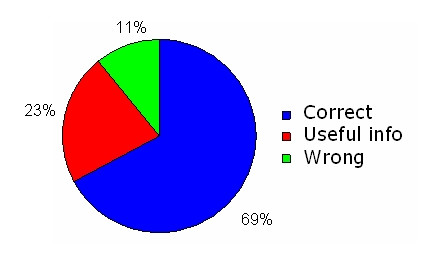
Pie chart describing the relative accuracy of the automated phylogenetic motif (PM) method using the {-1.0: -2.0} gap range. Assessment is determined using the structure-based approach described in [25]. The relative accuracy of the automated PM method is encouraging as it does not require any *a priori *structural information.

The quantitative assessment of functional site predictions from computational predictions remains an open problem in bioinformatics. Much of the difficulty arises from the fact that function and more specifically, functional sites, are ill defined concepts [38]. Aloy et al. [25] have attempted to standardize assessment of functional site predictions through comparisons to catalytically important residues (see Methods for technical details). Predicting functional sites with an automated evolutionary trace method [7] utilizing structural information on 86 protein families, Aloy et al. demonstrate impressive results: 79% correct, 15% useful information, and 6% Wrong. We use the same assessment strategy on 30 sampled protein families from the COG database (see Table 3). Fourteen of the analyzed families correspond to our earlier manual analysis [12] and the remaining are arbitrarily picked from examples with at least one solved structure. Figure 7 demonstrates that the PM functional site predictions are of similar quality to the overall accuracy reported by Aloy et al. Note that the dataset analyzed in Aloy et al. is not the same as the dataset analyzed here. This result is particularly encouraging due to the lack of structural details in the PM technique.

As Aloy et al. [25] point out, while the quality and robustness of SITE and ACTSITE records within PDB files is of varying quality, their approach does provide an automated and unbiased method for assessing functional site predictions. However, in automated efforts, examples of known "functional sites" will always be missed. For example, assessment of the TIM PM predictions indicates that 3 are correct, 4 provide useful information, and 1 is wrong. The "wrong" prediction actually corresponds to an evolutionarily conserved dimer interface epitope (see Figure 5) that includes several stabilizing monomer-monomer interactions [39]. Despite being far removed from the active site, binding of a small molecule at the dimer interface can inactivate the enzyme [40]. One of the three enzyme-inhibitor contacts occurs from Phe75, which is a residue within the "wrong" PM prediction. As a consequence, it could be argued that this PM is functional. This discussion is included here to encapsulate the ambiguity involved in functional site definitions and the difficulty in assessing their predictions.

## Conclusion

In this report, we present an automated algorithm which determines appropriate PSZ thresholds appropriate to functional site predictions. We demonstrate that our methodology is robust enough for large-scale analyses, while sensitive enough to identify known functional sites. For example, the method predicts all structural contacts, including the catalytic residue, between triosephosphate isomerase and its substrate. Additionally, the functionally important flexible "lid" is also identified. In the case of arginyl-tRNA synthetase, PMs correspond to regions surrounding both the amino acid/tRNA acceptor stem and enzyme-anticodon interactions. Using a quantitative structure-based functional site assessment algorithm, we demonstrate that the sequence-only PM predictions compare favorably to those from evolutionary trace approaches that are dependent upon solved structures.

## Availability and requirements

• **Project name: **MINER

• **Project home page: **

• **Operating system(s): **Platform independent

• **Programming language: **PERL

• **Other requirements: **Chime

• **License: **GNU GPL

• **Any restrictions to use by non-academics: **License needed

## List of abbreviations

Evolutionary trace (ET); Phylogenetic motif (PM); Phylogenetic similarity z-score (PSZ); Partition around medoids clustering (PAMC); Clusters of orthologous groups (COG); Triosephosphate isomerase (TIM).

## Authors' contributions

D. La was primarily responsible for the development and testing of the described methodology. D.R. Livesay oversaw the research. Both authors contributed equally to the writing of this manuscript.

## Figures and Tables

**Table 1:** Manual verses automatically determined thresholds^a^

**Protein family**	**PDB ID**	**Manual PSZ**	**Auto PSZ**
Acetyglucosamine-6-phoshate deacyetylase	1O12	-2.20	-2.25
Alcohol dehydrogenase	1JVB	-2.20	-2.15
CuZnSOD	1SPD	-1.50	-1.50
Cytochrome P450	1N4G	-2.20	-2.10
Enolase	2ONE	-2.00	-2.18
Glyceroladhyde-3-phosphate dehydrogenase	1DC4	-1.80	-1.90
Glycerol kinase	1B05	-2.00	-2.03
Glutamate dehydrogenase	1HWZ	-1.80	-2.12
Inorganic pyrophosphatase	1I6T	-1.80	-1.67
Myoglobin	1MBA	-1.50	-1.51
Succinate dehydrogenase – FAD	1NEK	-2.00	-2.20
Succinate dehydrogenase – Fe/S	1NEK	-2.20	-1.63
Succinate dehydrogenase – Heme	1NEK	-2.20	-1.88
TATA box binding protein	1TBP	-1.50	-2.16
Triosephosphate isomerase	7TIM	-1.50	-1.85

^a ^A sequence window width of 5 is used for each of the above examples and gap range of {-1: -2}.

**Table 2:** Correlation matrix of various phylogenetic motif parameters^a^

	**PSZ threshold**	**# phylogenetic motifs**	**# sequences**
**# phylogenetic motifs**	0.15		
**# sequences**	0.08	-0.07	
**MSA length**	-0.32	0.68	0.00

^a ^A sequence window width of 5 is used for each of the above examples and gap range of {-1: -2}.

**Table 3:** Structural assessment of the phylogenetic motif functional site predictions^a^

**Protein family**	**PDB ID**	**Correct**	**Useful info**	**Wrong**
*A. Proteins from Table 1*				

Acetyglucosamine-6-phoshate deacyetylase^b^	1O12	6	1	0
Alcohol dehydrogenase	1JVB	2	1	1
CuZnSOD	1SPD	3	2	1
Cytochrome P450	1N4G	6	4	0
Enolase	2ONE	6	2	0
Glyceroladhyde-3-phosphate dehydrogenase	1DC4	2	2	3
Glycerol kinase	1B05	7	1	0
Glutamate dehydrogenase	1HWZ	7	0	0
Inorganic pyrophosphatase	1I6T	3	2	0
Myoglobin	1MBA	4	1	0
Succinate dehydrogenase – FAD	1NEK	4	1	2
Succinate dehydrogenase – Fe/S	1NEK	4	0	0
Succinate dehydrogenase – Heme	1NEK	5	0	0
TATA box binding protein	1TBP	n/a^c^		
Triosephosphate isomerase	7TIM	3	4	1

*B. Additional examples*				

Acetate kinase	1G99	4	4	0
Aconitase A	7ACN	8	3	5
Alanine racemase	1L6F	4	1	1
Arginyl-tRNA synthetase	1F7U	2	2	1
Biotin carboxylase^b^	1DV2	3	3	2
Catalase	1GGF	9	0	0
Citrate synthase	1NXG	8	1	0
Isocitrate dehydrogenases	1IA2	3	0	0
Malate/lactate dehydrogenases	1EMD	6	1	0
Malate synthase	1P7T	6	0	0
Phosphomannomutase	1P5D	5	2	0
Selenocysteine lyase	1ECX	5	2	1
Threonine aldolase	1LW5	6	2	0
Thymidylate synthase	1AIQ	4	0	1
Transaldolase	1ONR	1	1	1
Trehalose-6-phosphate synthase	1UQU	5	3	3

^a ^A sequence window width of 5 is used for each of the above examples and gap range of {-1: -2}.

^b ^The known functional site defined in Acetyglucosamine-6-phoshate deacyetylase and biotin carboxylase is prohibitively incomplete; as such, functional sites indicated in [41] and [42], respectively, are also included. ^c ^Because so much of the TATA-box binding protein surface area directly interacts with its DNA substrate, the known functional site sphere encompasses nearly the entire protein, making the assessment unsporting because all predictions will trivially be correct.
